# Candidate genes for anthocyanin pigmentation in rice stem revealed by GWAS and whole‐genome resequencing

**DOI:** 10.1002/tpg2.20224

**Published:** 2022-06-15

**Authors:** Reza Haghi, Asadollah Ahmadikhah, Arash Fazeli, Vahid Shariati

**Affiliations:** ^1^ Leibniz Institute of Plant Genetics and Crop Plant Research (IPK) Seeland Germany; ^2^ Dep. of Plant Sciences and Biotechnology, Faculty of Life Sciences and Biotechnology Shahid Beheshti Univ. Tehran Iran; ^3^ Dep. of Agronomy and Plant Breeding Faculty of Agriculture Ilam Univ. Ilam Iran; ^4^ National Institute of Genetic Engineering and Biotechnology Tehran Iran

## Abstract

Anthocyanin pigment as a phenolic secondary metabolite is accumulated in areal organs of some rice cultivars. Despite several research attempts, the majority of genomic regions and candidate genes for purple‐colored stem (*Ps*) resulting from anthocyanin pigmentation of rice leaf sheath have not been identified. A genome‐wide association study (GWAS) and whole‐genome resequencing (WGR) analysis was applied for genetic dissection of anthocyanin pigmentation of rice stem. Using GWAS, the genomic regions (on chromosomes 2, 4, and 6) tagged to eight single‐nucleotide polymorphisms (SNPs) were identified to be significantly associated with purple stem, and in the vicinity of GWAS signals, 19 genes were highlighted as putative candidate genes. To narrow down the genomic regions more highly associated to the trait, a WGR study on recombinant inbred lines (RIL) with opposite phenotypes was conducted. After defining the DNA variation between reference genome, maternal parent and the two sister lines, a narrow genomic region on the short arm of chromosome 6 (4.7–6.2 Mbp interval) was identified to be highly associated with anthocyanin pigmentation of rice stem. In the interval, a few candidate genes with probable role in anthocyanin biosynthesis and accumulation were identified, which included five structural genes involved in the known pathways [one chalcone isomerase (CHI), two glycosyl transferases, and two UDP‐flavonoid‐3‐O‐glucosyl (UFGT) transferases] and two transcription factors [one basic helix–loop–helix (bHLH)‐ and one myeloblastosis (MYB)‐coding genes]. The identified candidate genes can be used in breeding programs of rice or other Gramineae species for anthocyanin accumulation in areal organs.

**Abbreviations**
bHLH: basic helix–loop–helix
CHI: chalcone isomerase
DFR: dihydroflavonol 4‐reductase
F3′H: flavonoid 3′‐hydroxylase
GWAS: genome‐wide association study
InDel: insertion–deletion
MBW complex: MYB–bHLH–WD40 repeats complex
MYB: myeloblastosis
RAPdb: Rice Annotation Project database
RIL: recombinant inbred line
UFGT: UDP‐flavonoid‐3‐O‐glucosyltransferase
WGR: whole‐genome resequencing

## 1 INTRODUCTION

Rice is a monocotyledonous plant belonging to the Gramineae family (Linares, 2002). Asian rice (*Oryza sativa* L.) is divided to three major ecological groups including *japonica*, *javanica*, and *indica* (Khush, 1997). The rice genome (2*n* = 2*x* = 24) has 436 Mbp length in haploid cells, smaller genome size, and fewer genes (40,000–50,000) than other cereals (Kurata et al., 2002). Small genome together with some other characteristics, including high rate of self‐pollination and easy genetic transformation, make rice as an appropriate model plant for cereals, and its genome has been successfully sequenced by collaboration of scientist from several countries in 2000 by shotgun approach (Eckardt, 2000).

**FIGURE 1:**
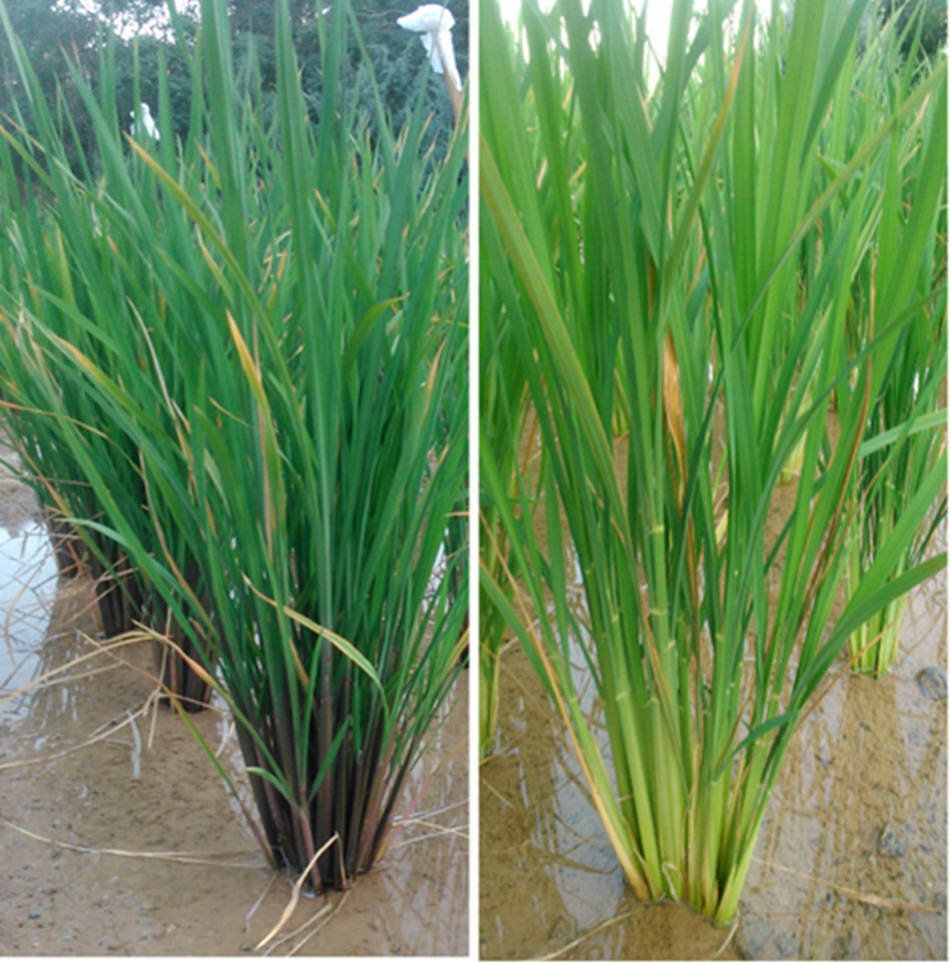
Anthocyanin pigmentation in purple‐colored stem cultivars (left) vs. green‐colored stem cultivars (right)

Because of anthocyanin pigment accumulation, some rice cultivars have purple areal organs instead of usual green color. The occurrence of purple pigment is more common in wild populations than in cultivars. Emergence of purple color begins from sheath of outer leaves in a few days after arising seedlings. The extension of the color is different in various cultivars; in some cultivars, it is remains restricted to leaf sheath but extends to all areal organs in other cultivars (Reddy et al., 1995). The outer leaf sheath of rice plant is called stem, the color of which may be in two forms: purple stem or green stem (Figure 1).

**Core Ideas**

- GWAS revealed a few genomic regions associated with anthocyanin pigment accumulation in rice stem.
- Using whole‐genome resequencing, a narrow interval on chr. 6 was determined to carry candidate genes for anthocyanin pigmentation in rice stem.
- Using recombinant inbred line sister lines in resequencing could filter out low‐power GWAS signals.
- Putative candidate genes on chromosome 6 include MYB, bHLH, CHI, glycosyl transferase, and UDP‐flavonoid‐3‐O‐glucosyltransferase.

**FIGURE 2:**
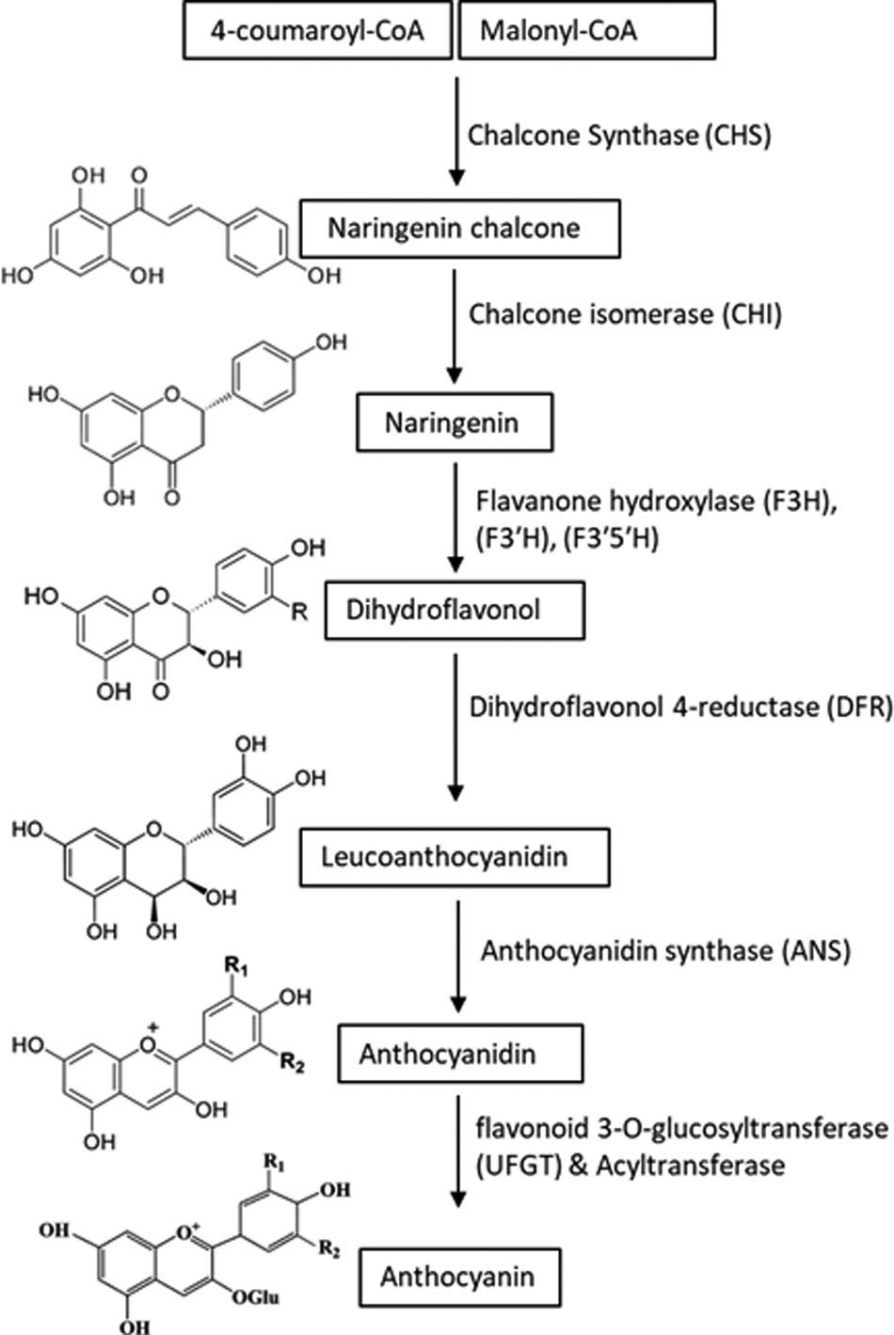
Biosynthesis pathway of anthocyanin in aerial organs of rice plant. The illustration was adopted from KEGG pathway database (http://genome.jp/kegg/)

Anthocyanin pigment, as a phenolic secondary metabolite, has several advantages for plants and humans including an important role in plant development; plant responses to biotic and abiotic stresses; the attraction of pollinator insects; antioxidant activity; and anticancer, hypoglycemic, and anti‐inflammatory effects (Petroni et al., 2014). Biosynthesis of anthocyanins branches from general flavonoid pathway by the activity of chalcone synthase enzyme converts 4‐coumaroyl‐CoA and malonyl‐CoA to nargenine chalcone (Figure 2). Then, chalcone isomerase (CHI) converts nargenine chalcon to nargenine. In the next step, three variants of dihydroflavonol can be synthesized by hydroxylase enzymes including flavanone 3‐hydroxylase, flavonoid 3′‐hydroxylase (F3′H), and flavonoid 3′,5′‐hydroxylase, which leads to purple anthocyanin pigmentation (Liu et al., 2018; Tanaka & Brugliera, 2013). Next, dihydroflavonol 4‐reductase (DFR) converts dihydroflavonol to a leucoanthocyanidin variant based on dihydroflavonol variant. Then, anthocyanidin synthase synthesizes different variants of a colored anthocyanidin from leucoanthocyanidins. Finally, attachment of glucose (sugar) by glucosyltransferase enzymes, for example by UDP‐flavonoid‐3‐O‐glucosyltransferase (UFGT), led to anthocyanin synthesis (Figure 2). The biosynthesis is processed in endoplasmic reticulum and then the synthesized anthocyanins are transferred to the vacuole through conjugating to glutathione with glutathione S‐transferase et al., 2014; Zheng et al., 2019). The synthesized anthocyanin might be further acylated by anthocyanin acyltransferase, which enhances stability of anthocyanin structures (Zheng et al., 2019; Petroni et al., 2014).

The synthesis of enzymes involved in the pathway such as flavonoid 3′,5′‐hydroxylase, DFR, anthocyanidin synthase, and UFGT are modulated by regulatory transcription factors, well known in maize (*Zea mays* L.) and other species (Chen, 2015; Gould et al., 2009). A regulatory complex consist of myeloblastosis (MYB), basic helix–loop–helix (bHLH), and WD40 repeats (MBW complex) are proposed as crucial and determinant modulator for final accumulation of anthocyanins in rice and other plants (Xu et al., 2015; Sun et al., 2018; Kim et al., 2018; Liu et al., 2018). The effect of B‐box, F‐box, and ELONGATED HYPOCOTYL 5 transcription factors on the expression of genes encoding the MBW complex have been reported as upstream regulators of the pathway in some rice tissues (Kim at al., 2018; Xia et al., 2016). Also, changes in activity and catalytic efficiency of structural genes of the pathway because of some amino acid substitution, and its effect on anthocyanin accumulation have been reported (Park et al., 2016). In previous studies, some various chromosomal regions were introduced as genomic region controlling anthocyanin accumulation in rice, but so far, no comprehensive study has been performed to identify the genes involved in this important trait. So, this study is dedicated to genes controlling anthocyanin biosynthesis in sheath leaf of rice plants by association mapping in a relatively large population and resequencing of sister lines prepared for this aim. Whole‐genome resequencing (WGR), which provides comprehensive and accurate information from various changes occurring in genomes, is often applied to identify single nucleotide polymorphisms (SNPs), copy number of variants, and structural changes in populations, as well as genome‐wide association study (GWAS) for identification of genes involved in morphological traits in well‐known genomes such as rice (Ponce et al., 2020; Huang et al., 2012). This method uses next‐generation‐sequencing tools for sequencing of DNA fragments and then applies a reference genome for assembling the fragments.

Linkage disequilibrium mapping, also called association mapping and GWAS, is a reliable method to identify the genomic regions involved in the control of quantitative and qualitative traits. Association mapping uncovers the correlation between marker alleles and the given trait in a natural prevalent population. In this method, a large and diverse collection of cultivars and lines is randomly collected, and the relevant quantitative trait loci are mapped based on linkage disequilibrium (Moose & Mumm, 2008). The natural population experienced a high rate of meiotic events and recombination through the evolution led to the high resolution of genetic maps and increases the accuracy of quantitative trait loci mapping (Zhang et al, 2016; Moose & Mumm, 2008). The efficiency of association mapping is highly dependent on coverage of the genome with genetic markers such as SPNs. The SNP genotyping has the advantage of presenting whole‐genome data without prohibitive cost, providing high‐resolution mapping with a large number of SNPs entire the genomes (Seo et al., 2020). However, the population structure and genetic relationship between different cultivars of the population can create false marker–trait associations. With the inclusion of population structure effect (*Q* coefficients) and kinship effects (*K* coefficients) to statistic model, the effect of population structure and kinship on the marker–trait association can be eliminated [Pritchard et al, 2000]. Accordingly, association mapping with a large number of cultivars and complete genome coverage with a large SNP data set (such as the 40 K SNP array used in this study) can identify all probable genes involved in the accumulation of anthocyanin pigments in rice. The objective of our research was to identify genomic regions involved in anthocyanin accumulation in rice and identification of candidate genes using association mapping with 40 K SNP array and compare the result with the WGR of recombinant inbred line (RIL) sister lines.

## 2 MATERIALS AND METHODS

### 2.1 Association mapping

The association panel including 282 rice accessions was received from the T. T. Chang Genetic Resources Center, International Rice Research Institute (Supplemental Table S1) and accessions were cultured in the research farm of Shahid Beheshti University (Tehran, Iran). The 40.0 K SNP array data for the same accessions (genotyped by Zhao et al. [2011]) was retrieved from the GRAMINE website (www.gramene.org). Phenotypic data for stem color (leaf sheath color, which was green‐ or purple‐colored) was recorded in field‐grown plants and converted to a 0 and 1 data matrix for subsequent analyses. Rice accessions were analyzed using STRUCTURE software v.2.3 based on Bayesian approach using SNP data (Pritchaard et al., 2000), and *Q* values representing the assignment of each accession to subgroups was extracted; also, the genetic relationship among accessions (e.g., kinship coefficients) was estimated using TASSEL 3.0 software (Bradbury et al., 2007). The two matrices, **Q** and **K**, were included in a mixed linear model as covariates. Then, association analysis between SNPs and stem color phenotype was conducted using TASSEL 3.0 software. The model is as follows:

$$\begin{array}{c} P=\mu +M+Q+K+E \end{array}$$

where **P** is the phenotype and **M** and **Q** are the genotypic fixed effects and population structure, respectively. To split the effect of genetic relationship between accessions, **K** matrix was added to the model as the kinship coefficient of the accessions, and **E** is the residual effects. After association analysis, the results were presented as Manhattan plots based on the negative log_10_ of *p* values (LOP) for each SNP–trait association. We used a threshold of *p* ≤ 1 × 10^−4^ (equal to LOP ≥ 4) to declare a significant association. The chromosome loci with high LOP values were considered as genomic regions carrying candidate genes. Based on the gene annotation database of rice (https://rapdb.dna.affrc.go.jp) and probable function of known genes in the vicinity of selected SNPs, candidate genes for the purple stem were identified.

### 2.2 Selecting of RIL lines for resequencing

A RIL population (consisted of 155 F_8_ lines) developed from a cross between ‘Neda’ (purple‐colored stem, *PsPs* genotype) × IR36 (green‐colored stem, *psps* genotype) was used in this research. The RIL population was segregated into green‐ and purple‐colored stem classes (*psps* and *Ps‐*) as expected. In spite of the self‐pollination in each generation (from F_2_ to F_8_) and expectance to be inbred across most of genetic loci, few RIL lines with dominant phenotype (purple‐colored stem) showed remaining heterozygosity, as they showed segregation for stem color when their seeds were cultured in separate rows in the next growing season (offspring test). This phenomenon was explored for genetic dissection of *Ps* locus and identification of putative candidate genes at the locus by using DNA WGR. For this, a single, purple‐colored stem F_8_ RIL line (RIL line #78 with *Psps* genotype) was selected, and its F_9_ offspring seeds were cultured next season. After emerging, the stem color phenotype at early tillering stage, the offspring of line #78, were visually inspected to distinguish stem color, and the seeds of the F_9_ sister lines (offspring of RIL line #78) with opposite stem colors were collected and stored till offspring test at next season. Next season, the seeds of single F_10_ sister lines were cultured in separate rows. The segregating rows were excluded, and only two inbred RIL lines with opposite phenotypes were selected: a single F_10_ sister line with green‐colored stem (line RC5 of *psps* genotype) and a single F_10_ sister line with purple‐colored stem (line RC10 of *PsPs* genotype). These two F_10_ sister lines along with the maternal parent with purple‐colored stem (Neda) were used for WGR analysis. Comparing the sequenced genomes of the sister lines with opposite stem colors along with their maternal parent led to the identification of genomic region affecting accumulation of anthocyanin in stem of rice plant.

### 2.3 WGR

The genomic DNA of Neda cultivar (purple‐colored stem) and the two sister lines with opposite stem color were extracted using the method of Ahmadikhah (2009). The quality and quantity of extracted DNA were surveyed using spectrophotometer and agarose gel. Whole‐genome resequencing of the samples were performed by Illumina HiSeqTM2000 platform (BGI, China) with the paired‐end method.

The output of sequencing was obtained as short reads in .fastq format. In the first step, the quality of sequences was surveyed using FASTQC software for sequencing quality parameters including per‐base sequencing quality, per‐sequence quality scores, per‐base sequence content, per‐sequence glycine and cysteine content, adaptor contamination, among others. After ensuring the quality of sequences, the low‐quality reads (Q30 < 20), reads with adaptor contamination, and duplicated reads were filtered using Trimmomatic software. As the rice samples (purple parent and two sister lines) used in this study had *indica* background genome, the Oryza_indica.ASM465v1 reference genome obtained from Ensembl database (https://plants.ensembl.org) was used for alignment mapping. Next, to do assembly of the short reads, the filtered reads were aligned to the reference genome using MEM algorithm in Burrows–Wheeler Aligner software. Next, to simplify use of the alignment output file, the file size was reduced by converting the format from SAM (human‐readable text files) to BAM (binary alignment equivalent), and then, all the reads were sorted based on their genomic position in SAMtools software. Afterward, the duplicated reads were identified and removed by MarkDuplicates tools in Picard software. During the alignment process, some small missarrangements may occur in the genomic region around the insertion–deletion (InDel) markers; to correct this, likely mistakes of the map, the reads around the InDel markers were realigned by GATK software. The quality of mapping was evaluated based on calculating the average sequencing depth and coverage by the SAMtools in the alignment results.

After controlling the quality of mapping, mapped reads were used to identify the SNPs and InDel markers via local de novo assembly of haplotypes using HaplotypeCaller algorithm in GATK pipeline. Then, polymorphisms (the SNP and InDel markers) were annotated based on the annotation of reference genome databases using SnpEff software, and genomic location, hetero‐ or homozygosity, and other details of polymorphisms were described. The outputs were transferred to Excel environment, and the SNP and InDel markers were queued based on their genomic location for haplotype form of both resequenced lines and reference genome. It is notable that the reference genome (line 9311) has green‐colored stem. Knowing the reference genome has green‐colored stem, the called SNP variants should be the same in the green‐colored RIL (sister line RC5) and reference genome and should be different from the two purple‐colored stem samples (including sister line RC10 and the maternal parent Neda); on the other hand, the SNP variants should be the same in both samples with purple‐colored stem. So, firstly the heterozygote variants and the variants that were different between samples with same phenotype were filtered. Then, the variants that were identical between samples with different phenotype were omitted. Finally, the remained SNPs were considered as true variants (they were different between samples with different phenotype and identical between the samples with the same phenotype). These SNP and InDel markers are supposed to likely mark the genes involved in anthocyanin pigment accumulation in purple‐colored stems of the rice plant. The sequences of this study were deposited in the short read archive database under accession numbers SAMN24114689, SAMN24114690, and SAMN24114691.

The list of annotated genes for rice plants were downloaded from the Rice Annotation Project database (RAPdb, https://rapdb.dna.affrc.go.jp). In this list, based on genomic position of detected SNP and InDel polymorphisms, we traced and focused on the genes that were annotated as putative genes of anthocyanin accumulation.

## 3 RESULTS

### 3.1 Association mapping study

**FIGURE 3:**
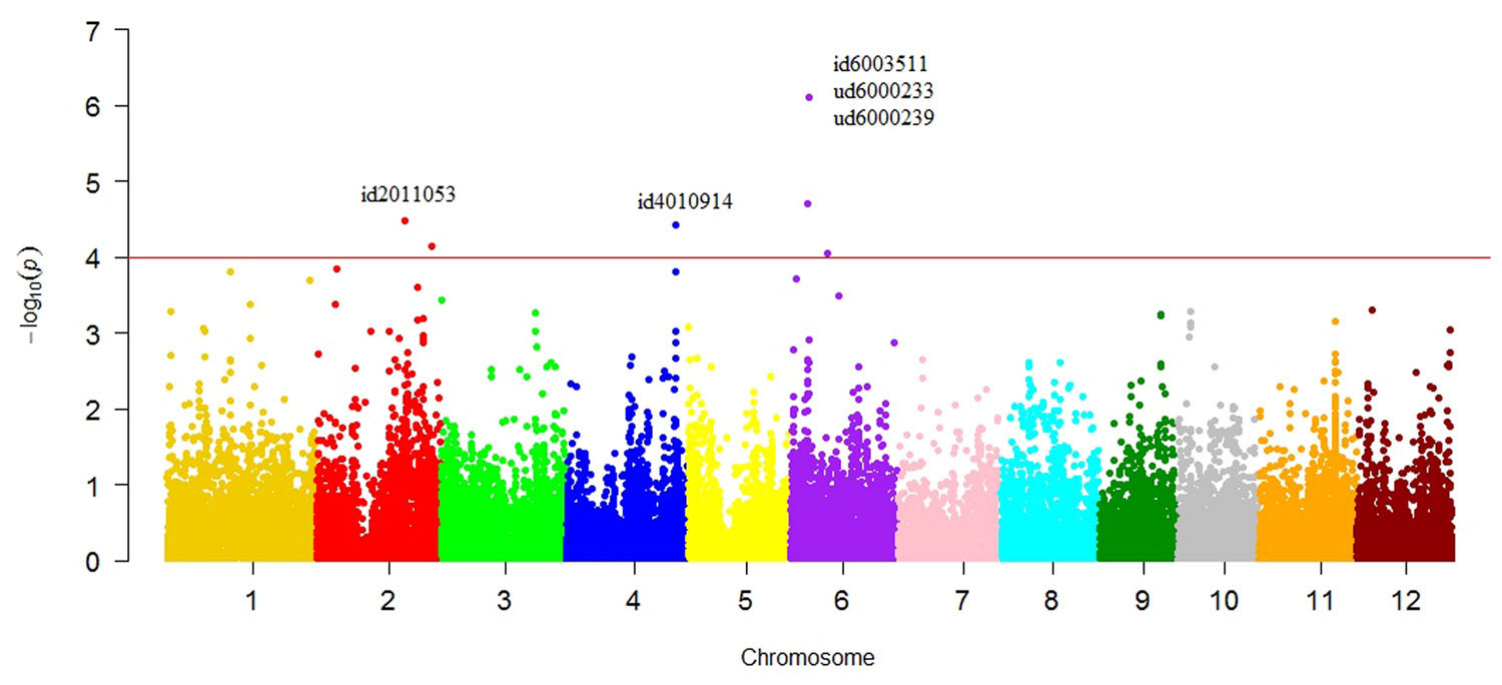
Genome‐wide association study analysis results for the anthocyanin pigments accumulation in purple stems of rice plants. Chromosomes were shown with different colors. Identification numbers of leader single‐nucleotide polymorphisms above red line threshold (negative log_10_ (*p*) > 4) were depicted

The results of GWAS revealed that eight SNPs on chromosomes 2, 4, and 6 were significantly associated with anthocyanin pigmentation (Figure 3). Based on the RAPdb, in the vicinity of the significant SNP signals (e.g., up to 1 Mbp in both directions), 19 genes were identified as being likely involved in purple stem pigmentation including: six MYB, four bHLH, two WD40 repeats, two CHI, one B‐Box, two glucosyl transferases, and two UDP‐glucosyl transferases (Table 1). Interestingly, three SNPs with a strong signal (−log_10_(*p*) > 6) were detected in a narrow interval on the short arm of chromosome 6 (from 5,295,675 to 5,351,422 bp). In the vicinity of this genomic region, seven annotated genes reside including two MYB (*Os06g0205100* and *Os06g0221000*), one CHI (*Os06g0203600*), two glucosyl transferases (*Os06g0212300* and *Os06g0212400*), and two UDP‐glucosyl transferases (*Os06g0216100* and *Os06g0220500*).

**TABLE 1:** Candidate genes identified in association study which putatively involved in anthocyanin pigmentation in rice purple‐colored stems

Chromosome	SNP position	LOP	Candidate genes in vicinity of associated SNP	Candidate gene position	Description of candidate genes
	bp			bp	
2	25,401,253	4.47	*Os02g0618400*	24,580,457–24,582,527	MYB coding gene
			*Os02g0624300*	24,878,777–24,879,932	MYB coding gene
			*Os02g0641300*	25,759,841–25,762,800	MYB coding gene
			*Os02g0641900*	25,778,113–25,780,795	MYB coding gene
			*Os02g0646200*	26,027,785–26,029,488	B‐box coding gene
	33,022,850	4.14	*Os02g0770100*	32,469,587–32,472,658	WD40 repeat domain coding gene
			*Os02g0778500*	32,957,456–32,961,345	CHI coding gene
			*Os02g0791800*	33,629,573–33,633,614	WD40 repeat domain coding gene
4	31,715,352	4.43	*Os04g0618600*	31,416,378–31,420,095	bHLH coding gene
			*Os04g0631600*	32,166,724–32,169,575	bHLH coding gene
			*Os04g0641700*	32,657,307–32,658,183	bHLH coding gene
6	5,058,807	4.70	*Os06g0193400*	4,709,744–4,715,552	bHLH coding gene
	5,295,675	6.10	*Os06g0203600*	5,227,416–5,231,610	CHI coding gene
	5,295,818	6.10	*Os06g0205100*	5,315,163–5,316,640	MYB coding gene
			*Os06g0212300*	5,718,733–5,720,698	Glycosyl transferase coding gene
			*Os06g0212400*	5,724,872–5,726,745	Glycosyl transferase coding gene
	5,351,422	6.10	*Os06g0216100*	5,908,095–5,909,128	UDP‐glucosyl transferase coding gene
			*Os06g0220500*	6,205,613–6,207,557	UDP‐glucosyl transferase coding gene
			*Os06g0221000*	6,239,975–6,241,172	MYB coding gene
	10,550,728	4.04	*–*	–	–

### 3.2 Resequencing study

**FIGURE 4:**
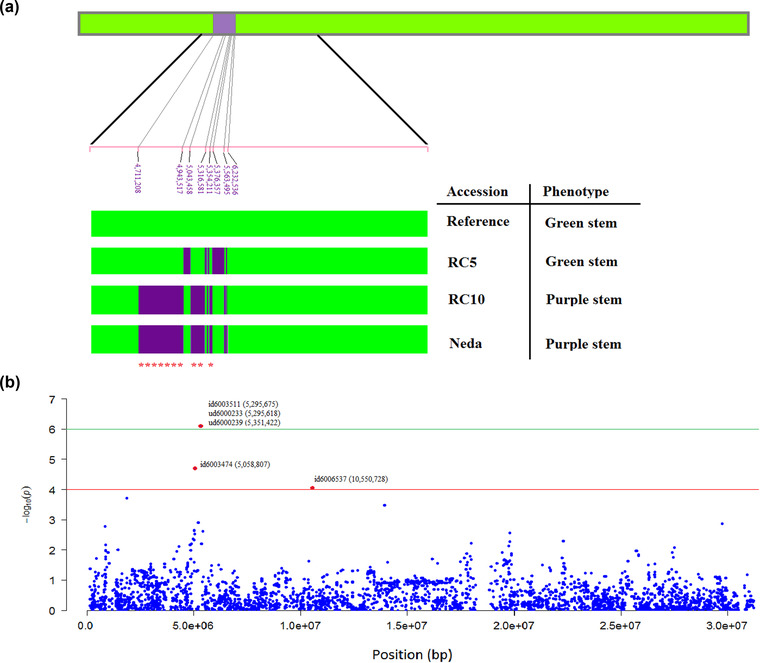
Comparison of resequencing and genome‐wide association study (GWAS) analyses for chromosome 6. (a) Variable regions between accessions with green‐colored and purple‐colored stems based on alignment of resequenced genomes. The most probable candidate intervals were marked with red asterisks. (b) GWAS result for purple‐colored stem as obtained using single‐nucleotide polymorphisms (SNPs) on chromosome 6. SNPs and their positions (in parentheses) with higher association were shown

After alignment of the resequenced genomes with the reference genome and applying the appropriate filters mentioned in the Material and Methods section, the remained DNA variations included 1,482 SNP and InDel markers that could be tentatively assumed as being correlated with anthocyanin pigmentation in purple stems. Interestingly, 1,448 out of the 1,482 variations were densely located in a narrow interval on short arm of chromosome 6 from 4.711 to 6.232 Mbp, and the rest of the 34 polymorphisms were scattered on other chromosomes (Figure 4). This result is compatible with the chromosomal region identified by GWAS analysis (5.058–5.351 Mbp on chromosome 6). Regarding the genomic structure of this region and considering the recombination events, candidate intervals include 4.711–4.943, 5.043–5.316, and 5.354–5.376 Mbp (the intervals shown with red asterisks in Figure 4a). This region is well validated by GWAS analysis (Figure 4b). Though, in the mentioned region of chromosome 6 (4.711–6.232 Mbp) based on surveying the list of RAPdb, seven annotated genes with a higher probable role in controlling the anthocyanin pigmentation were found that included two transcription factors and five structural genes (Table 2). Among the structural genes involved in the known pathways, one CHI, two glycosyl transferases, and two UFGTs can be highlighted (Table 2). Among the candidate regulatory genes, one bHLH and one MYB coding genes were detected in the genomic region. As said, 34 DNA variations (SNP and InDel markers) were distributed on other chromosomes and any of the putative candidate genes (listed in Supplemental Table S2 according to literature review) were not found in the vicinity of these variations. Thus, we can conclude that they resulted from the remaining heterozygosity in sister lines and do not have correlation with the purple pigmentation of stem.

**TABLE 2:** Candidate genes on chromosome 6 of rice identified in resequencing study that are probably involved in anthocyanin pigmentation of rice purple stems

Candidate gene	Position on chromosome 6	Gene description	Validated by GWAS?
	bp		
*Os06g0193400*	4,709,744–4,715,552	bHLH coding gene	Yes
*Os06g0203600*	5,227,416–5,231,610	Chalcone isomerase coding gene	Yes
*Os06g0205100*	5,315,163–5,316,640	MYB coding gene	Yes
*Os06g0212300*	5,718,733–5,720,698	Glycosyl transferase coding gene	Yes
*Os06g0212400*	5,724,872–5,726,745	Glycosyl transferase coding gene	Yes
*Os06g0216100*	5,908,095–5,909,128	UDP‐flavonoid‐3‐O‐glucosyl transferase coding gene	Yes
*Os06g0220500*	6,205,613–6,207,557	UDP‐flavonoid‐3‐O‐glucosyl transferase coding gene	Yes

## 4 DISCUSSION

The highest −log_10_ (*p*) of GWAS signals were located in a narrow region (∼300 Kbp) on short arm of chromosome 6 including three SNPs with −log_10_ (*p*) > 6 and one SNP with −log_10_ (*p*) = 4.7. Interestingly, the region is completely located inside the region showing highest genomic variation in the resequencing study (Figure 3). In spite of using different plant material (a diverse germplasm of 280 cultivars in GWAS and RIL lines in resequencing study), the compatibility of the results of association and resequencing studies strongly indicates the accuracy and validation of the applied methods for genetic dissection of purple‐colored stem phenotype. Using two RIL sister lines with high genomic similarity (as revealed by resequencing analysis in which only 1,482 DNA variants were detected) could filter out the low‐power GWAS signals and resulted in narrowing down of the responsive genomic regions for the trait under study.

Based on previous studies in rice and other plant species, 11 structural genes and transcription factor families were considered as probably responsible genes for the accumulation of anthocyanin pigments in plant organs (Supplemental Table S2). The MBW complex is the mostly accepted regulatory complex that modulates the expression of the structural genes in the respective pathway in various genera. The MYB component is the main enzyme of the complex, binding to promoter region of the target gene to regulate its expression. The bHLH provides the specificity of complex in recognition of the target genes, and WD40 increases the stability of the complex (Liu et al., 2018). Both MYB and bHLH coding genes were detected in our candidate interval carrying genomic variations in resequenced samples (Figure 3; Table 2).

Glycosylation is final determiner of anthocyanins variants by concatenating various sugar groups to anthocyanidins and has an important role in stabilization and further adjustments such as acylation and transportation of colored anthocyanins (Sasaki et al., 2014; Cheng et al, 2014). One or more of the four detected glycosyl transferase genes within the variable resequenced region seems to be the glycosyl transferase encoding genes involving in anthocyanin biosynthesis.

The structural genes involved in the anthocyanins’ biosynthesis encode DFR, flavonoid 3′,5′‐hydroxylase, chalcone synthase, and CHI, which are well characterized in various species and the allelic variation leading to alter the final anthocyanin accumulation have been reported for some of these genes (Liu et al., 2018). For instance, Park et al. (2016) reported allelic variation in F3′H genes (leading to one or two amino acid substitutions) from white, black, and red rice. The catalytic activity of F3′H enzyme derived from black rice showed two‐fold increase compared with red and white rice samples. The MBW complex as an accepted regulatory complex modulating the expression of the structural genes in the pathway (Liu et al., 2018) and previous studies on the MBW complex suggested that the expression of the MYB and bHLH regulatory genes is specific for pigmented tissue in most cases, while expression level of WD40s, which are involved in stabilizing the MBW complex, is generally similar between anthocyanin‐pigmented and nonpigmented tissues (Ramsay & Glover, 2005; Li, 2014; Wang et al., 2019). In this study, both MYB and bHLH genes were placed in a genomic region controlling purple stem phenotype by both GWAS and resequencing analyses, and hence we suggest that the two genes encoding MYB and bHLH (*Os06g0205100* and *Os06g0193400*) residing in the variable region of chromosome 6 (4.7–6.2 Mbp interval) can be considered as members of MBW complex although evidence for involvement of the first gene in regulation of anthocyanin pigmentation of rice stem are more reliable. The first gene was named the rice homologue of maize C1 (Reddy, 1998) and it was related to different phenotypes including colored apiculus and stigma (Saitoh et al., 2004) and purple leaf sheath (Chin et al., 2016). The gene was annotated as R2R3‐MYB, transcription factor *MYB6*, *OsC1* in RAPdb (http://rapdb.dna.affrc.go.jp/). The MYB gene regulates other genes involved in anthocyanin synthesis and enhancing another transcription factor, bHLH, regulating anthocyanin structural gene *DFR* (Dooner et al., 1991; Sainz et al., 1997; Ithal & Reddy, 2004; Li et al., 2017). As reported, the *OsC1* MYB interacts with stress‐responsive promoter elements of *OsDfr* and *OsAns* (flavonoid pathway genes) to induce tolerance to dehydration, high salt, and abscisic acid (Ithal & Reddy, 2004). The R2R3‐MYB transcription factors participate in regulating flavonoid biosynthesis in plants particularly in production of anthocyanin and proanthocyanin (Upadhyaya et al., 2021). The latter gene (*Os06g0193400*) was annotated as *OsbHLH096*, inorganic phosphate starvation‐induced transcription factor 1, *OsPTF1*, in RAPdb. The gene introduced as a transcription factor involved in tolerance to phosphate starvation in rice (Yi et al., 2005) and, to date, there is no evidence of its involvement in regulation of anthocyanin biosynthesis; hence, it is open for further investigation of its probable regulatory role in anthocyanin biosynthesis. Although, the involvement of *GMYC1* (bHLH transcription factor) in regulation of gerbera *DFR* was reported (Elommaa et al., 1998), *OsbHLH096* has no significant polypeptide and DNA sequence similarity with *GMYC1* (data not shown), and hence, it is different from *GMYC1*, thus functional analysis is needed to elucidate its role in molecular and biological processes such as anthocyanin pigmentation of rice aerial organs.

## 5 CONCLUSION

In this study, a few genomic regions were identified to be associated with anthocyanin pigment accumulation in rice stem. Using RIL sister lines in WGR, low‐power GWAS signals were filtered out and only a narrow interval on chromosome 6 was determined to carry candidate genes for anthocyanin pigmentation in rice stem. In this interval, putative candidate genes on chromosome 6 included MYB, bHLH, CHI, glycosyl transferase, and UFGT. The identified candidate genes can be used for metabolite engineering of rice and also in breeding programs of rice or other Gramineae species for regulating anthocyanin accumulation in areal organs, and in the case of some candidate genes, they may be useful for tolerance to oxidative stress.

## AUTHOR CONTRIBUTIONS

Reza Haghi: Formal analysis; Visualization; Writing – original draft. Asadollah Ahmadikhah: Conceptualization; Funding acquisition; Investigation; Project administration; Resources; Supervision; Visualization; Writing – review & editing. Arash Fazeli: Investigation; Project administration; Writing – review & editing. Vahid Shariati: Methodology; Software; Writing – review & editing.

## CONFLICT OF INTEREST

The authors declare that there is no conflict of interest.

## Supporting information


**Supplemental Table S1**. The rice accessions used in the study.

**Supplemental Table S2**. Candidate genes involved in anthocyanin pigment accumulation in plant organs identified in previous studies.
