# Supplementary material for: Candidate genes for anthocyanin pigmentation in rice stem revealed by GWAS and whole‐genome resequencing
Source: Plant Genome. 2022 Jun 15;15(3):e20224. doi: 10.1002/tpg2.20224 (PMC12807308; doi:10.1002/tpg2.20224)
Supplement: Supplementary file 1 [file TPG2-15-e20224-s001.docx]

**Supplementary Table S2.** Candidate genes involved in anthocyanin pigment accumulation in plant organs identified in previous studies.

| **Genes** | **Abbreviation** | **Functional class** | **Reference** |
| --- | --- | --- | --- |
| Basic helix-loop-helix gene | bHLH | transcription factor | Nesi et al. (2000), Sakamoto et al. (2001),  Xu et al. (2015) |
| Myb DNA-binding domain coding gene | MYB | transcription factor | Hartmann et al. (1998), Upadhyaya et al. (2021), Xu et al. (2015) |
| WD40 repeat domain coding gene | WDR | transcription factor | Xu et al. (2015) |
| B-BOX domain coding gene | B-Box | transcription factor | Kim et al. (2018) |
| F-Box domain coding gene | F-Box | transcription factor | Xia et al. (2016) |
| Flavonoid 3′,5′-hydroxylase | F3′5′H | structural gene | Falginella et al. (2010) |
| Dihydroflavonol 4-reductase | DFR | structural gene | Nakai et al. (1998), Furukawa et al. (2007), Li et al. (2017) |
| Chalcone synthase | CHS | structural gene | Reddy et al. (1996) |
| Chalcone isomerase | CHI | structural gene | Druka et al. (2003) |
| Anthocyanidin synthase | ANS | structural gene | Reddy et al. (2007) |
| Acyltransferase | AT | structural gene | Murayama et al. (2021) |
| flavonoid 3-O-glucosyltransferase | UFGT | structural gene | Peng et al. (2017) |
